# Supplementary material for: Social state alters vision using three circuit mechanisms in Drosophila
Source: Nature. 2024 Nov 20;637(8046):646–53. doi: 10.1038/s41586-024-08255-6 (PMC11735400; doi:10.1038/s41586-024-08255-6)
Supplement: Supplementary file 1 — Supplementary Tables 1–37. [file 41586_2024_8255_MOESM1_ESM.docx]

**Supplementary Table 1: Key Resource Table**

| REAGENT or RESOURCE | SOURCE | IDENTIFIER |
| --- | --- | --- |
| Experimental models: Organisms/strains | | |
| *D. melanogaster: w^1118^;; 20XUAS-CsChrimson-mVenus (su(Hw)attP1)* | Klapoetke et al., 2014;  Aso et al., 2014 | Chrimson |
| *D. melanogaster: w^1118^; BPp65ADZp (attP40); BPZpGAL4DBD (attP2)* | Pfeiffer et al., 2010;  Aso et al., 2014; Hampel et al., 2015 | Empty-SS |
| *D. melanogaster: w^1118^; VT064565-p65ADZp (attP40); VT043699-ZpGAL4DBD (attP2)* | Schretter et al., 2020 | aIPg-SS; SS36564  Available via https://www.janelia  .org/split-GAL4. |
| *D. melanogaster: norpA^36^; Sp/CyO; TM6B/TM2* | Longden et al., 2023 | NorpA^-^ |
| *D. melanogaster: w^1118^;; 20XUAS-GtACR1-EYFP (attP2)/TM2* | Barry Dickson/Ryo Minegishi | GtACR |
| *D. melanogaster: w^1118^; LexAop-TRPA1 (VIE-260B); 20XUAS-GtACR1-EYFP (attP2), R72C11-iLexA (VK00005)* | This study | aIPg-LexA > TrpA  GtACR |
| *D. melanogaster: w^1118^; R28G03-p65ADZp (attP40); VT027704-ZpGL4DBD (attP2)* | Wu, Nern et al., 2016 | LC9-SS; SS02662  Available via https://www.janelia  .org/split-GAL4. |
| *D. melanogaster: w^1118^; R35D04-p65ADZp (attP40); R22D06-ZpGAL4DBD (attP2)* | Wu, Nern et al., 2016 | LC10a-SS; OL0019B  Available via https://www.janelia  .org/split-GAL4. |
| *D. melanogaster: w^1118^; R35D04-p65ADZp (attP40); VT040747-ZpGAL4DBD (attP2)* | Wu, Nern et al., 2016 | LC10bc-SS; SS02663  Available via https://www.janelia  .org/split-GAL4. |
| *D. melanogaster: w^1118^; R22H02-p65ADZp (attP40); R20G06-ZpGAL4DBD (attP2)* | Wu, Nern et al., 2016 | LC11-SS; OL0015B  Available via https://www.janelia  .org/split-GAL4. |
| *D. melanogaster: w^1118^; R26A03-p65ADZp (attP40); R24A02-ZpGAL4DBD (attP2)* | Wu, Nern et al., 2016 | LC15-SS; OL0042B  Available via https://www.janelia  .org/split-GAL4. |
| *D. melanogaster: w^1118^; R59B10-p65ADZp (attP40); R24A02-ZpGAL4DBD (attP2)/TM6B* | This study | IB112-SS1; SS81529  Available via https://www.janelia  .org/split-GAL4. |
| *D. melanogaster: w^1118^; R59B10-p65ADZp (JK22C), R24A02-ZpGAL4DBD (attP2)/TM6B* | This study | IB112-SS2; SS81571  Available via https://www.janelia  .org/split-GAL4. |
| *D. melanogaster: w^1118^, 13XLexAop2-CsChrimson-tdTomato (attP18); R72C11-iLexA (JK22C); pJFRC81-10XUAS-IVS-Syn21-GFP-p10 (attP2)* | This study | aIPg-LexA > Chrimson  GFP |
| *D. melanogaster: w^1118^, 13XLexAop2-CsChrimson- tdTomato (attP18);; pJFRC81-10XUAS-IVS-Syn21-GFP-p10 (attP2)* | This study | LexAop-Chrimson  GFP |
| *D. melanogaster: w^1118^; VT061221-p65ADZp (attP40); R18C12-ZpGAL4DBD (attP2)* | This study | TuTuA_1-SS; SS77402  Available via https://www.janelia  .org/split-GAL4. |
| *D. melanogaster: w^1118^; R21H09-p65ADZp (attP40); R34C04-ZpGAL4DBD (attP2)* | This study | TuTuA_2-SS1; SS77462  Available via https://www.janelia  .org/split-GAL4. |
| *D. melanogaster: w^1118^; R21H09-p65ADZp (attP40); VT016645-ZpGAL4DBD (attP2)* | This study | TuTuA_2-SS2; SS101666  Available via https://www.janelia  .org/split-GAL4. |
| *D. melanogaster: w^1118^, 20XUAS-CSChrimson-tdTomato (attP18); pJFRC57-13XLexAop2-IVS-GFP-p10 (su(Hw)attP5); SL96683 (VK00027, VK00005)* | This study | TuTuA_2-LexA > GFP  Chrimson; SL96683  Available via https://www.janelia  .org/split-GAL4. |
| *D. melanogaster: w^1118^; R35C10-p65ADZp (JK73A), R71A09-ZpGAL4DBD (attP2)* | Schretter et al., 2020 | pC1d-SS; SS56987  Available via https://www.janelia  .org/split-GAL4. |
| *D. melanogaster: w^1118^; R35C10-p65ADZp (JK73A), R60G08-ZpGAL4DBD (attP2)* | Schretter et al., 2020 | pC1e-SS; SS59336  Available via https://www.janelia  .org/split-GAL4. |
| *D. melanogaster: w^1118^; VT025602-p65ADZp (attP40); VT002064-ZpGAL4DBD (attP2)/TM6B* | Schretter et al., 2020 | pC1d/e-SS; SS43274  Available via https://www.janelia  .org/split-GAL4. |
| *D. melanogaster: w^1118^, 13XLexAop2-CsChrimson-tdTomato (attP18), 20XUAS-IVS-Syn21-opGCaMP6f-p10 (su(Hw)attP8);; R72C11-LexAi (VK00005)/TM6B* | This study | aIPg-LexA > Chrimson  GCaMP6f |
| *D. melanogaster:* *w^1118^, 13XLexAop2-CsChrimson-tdTomato (attP18), 20XUAS-IVS-Syn21-opGCaMP6f-p10 (su(Hw)attP8); VT029314-LexA (JK22C)/CyO; +/TM6B* | This study | LC10a-LexA > Chrimson  GCaMP6f |
| *D. melanogaster:* *w^1118^, 13XLexAop2-CsChrimson-tdTomato (attP18), 20XUAS-IVS-Syn21-opGCaMP6f-p10 (su(Hw)attP8); +/CyO; +/TM6B* | This study | LexAop-Chrimson  GCaMP6f |
| *D. melanogaster: w^1118^, 20XUAS-CsChrimson-mVenus (attP18); LexAop-TRPA1 (VIE-260B); R72C11-iLexA (VK00005)* | This study | aIPg-LexA > TrpA Chrimson |
| *D. melanogaster:* *w^1118^;; 20XUAS-IVS-jGCaMP7f (VK00005)* | Dana et al., 2019; Hindmarsh Sten et al., 2021 | GCaMP7f |
| Software and algorithms | | |
| Matlab R2019a | Mathworks | https://www.mathworks.com |
| ImageJ 2.1.0 | Schneider et al., 2012 | https://imagej.nih.gov/ij/ |
| Caltech FlyTracker | Eyjolfsdottir, 2017 | https://github.com/kristinbranson/FlyTracker |
| JAABA | Kabra et al., 2013 | https://jaaba.sourceforge.net |
| FlyDisco Analysis Pipeline | Robie et al., 2024 | https://github.com/kristinbranson/FlyDiscoAnalysis |
| VVD viewer |  | https://github.com/JaneliaSciComp/VVDViewer |

**Supplementary Table 2: Behavioral Classifiers Table**

| **Aggression and Touch JAABA Classifiers.** | |  |  |  |
| --- | --- | --- | --- | --- |
|  | Framewise Performance | |  |  |
| Classifier | True Positive | True Negative | False Positive | False Negative |
| Aggression | 89.5% (2072) | 98.4% (2515) | 10.5% (244) | 1.6% (40) |
| Touch | 97.4% (1939) | 86.7% (1818) | 2.6% (52) | 13.3% (278) |

**Supplementary Tables 3 – 37: Genotypes, Sample sizes, and Statistics**

For genotypes, group name used in figures is listed under group title along with simplified names used under condition title in statistics tables.

For statistical analysis of optogenetic experiments, both a Wilcoxon Matched-pairs Signed Rank test (two-tailed) and a Two-way ANOVA with a Tukey test (three or more groups) or Uncorrected Fisher’s LSD (two groups) for multiple comparisons were used and are reported below. See Source Data excel file for values used in calculations.

**Supplementary Table 3: Genotypes for Figure 3a**

| Genotype | Cells Imaged/Indicator | Stable split name |
| --- | --- | --- |
| *w^+^(DL)/ w^1118^; +/R28G03-p65ADZp;20XUAS-IVS-Syn21-GCaMP6f-p10/ VT027704-ZpGAL4DBD* | LC9/GCaMP6f | SS02662 |
| *w^+^(DL)/ w^1118^; +/ R35D04-p65ADZp; 20XUAS-IVS-Syn21-GCaMP6f-p10/ R22D06-ZpGAL4DBD* | LC10a/GCaMP6f | OL0019B |

**Supplementary Table 4: Genotypes for Figure 3d**

| Genotype | Group |
| --- | --- |
| *Canton-S* | Courtship (Male-Female) |
| *w^1118^; LexAop-TRPA1/ BPp65ADZp; 20XUAS-GtACR1-EYFP, R72C11-iLexA/ BPZpGAL4DBD* (aIPg-LexA > TrpA empty-SS > GtACR) | Aggression (Female) |

**Supplementary Table 5: Genotypes for Figure 3e – f**

| Genotype | Group |
| --- | --- |
| *w^1118^; LexAop-TRPA1/ BPp65ADZp; 20XUAS-GtACR1-EYFP, R72C11-iLexA/ BPZpGAL4DBD* | aIPg > TrpA empty > GtACR (empty group) |
| *w^1118^; LexAop-TRPA1/ VT064565-p65ADZp; 20XUAS-GtACR1-EYFP, R72C11-iLexA/ VT043699-ZpGAL4DBD* | aIPg > TrpA aIPg > GtACR (aIPg group) |
| *w^1118^; LexAop-TRPA1/ R35D04-p65ADZp; 20XUAS-GtACR1-EYFP, R72C11-iLexA/ R22D06-ZpGAL4DBD* | aIPg > TrpA LC10a > GtACR (LC10a group) |
| *w^1118^; LexAop-TRPA1/ R35D04-p65ADZp; 20XUAS-GtACR1-EYFP, R72C11-iLexA/ VT040747-ZpGAL4DBD* | aIPg > TrpA LC10bc > GtACR (LC10bc group) |

**Supplementary Table 6: Sample sizes and Statistics for Figure 3e – f**

Analysis – Average percent of time spent performing aggressive behaviors during 15s before the first stimulus period compared to the average of the three 30s continuous stimulus (9 mW/cm^2^) periods.

Each experiment is a biological replicate and data are combined from 6 batches from independent parental crosses. Negative controls were performed with each batch.

| Figure | Condition | # | Sample Size | Statistical  Test | Comparison | P-value |
| --- | --- | --- | --- | --- | --- | --- |
| 3f | Empty group –  Pre-stimulus | 1 | 26 | Wilcoxon  Matched-pairs | 1 v 2 | 0.6171 |
|  | Empty group –  Stimulus  aIPg group –  Pre-stimulus  aIPg group –  Stimulus  LC10a group –  Pre-stimulus  LC10a group –  Stimulus  LC10bc group –  Pre-stimulus  LC10bc group –  Stimulus | 2  3  4  5  6  7  8 | 26  26  26  23  23  23  23 | Signed Rank  (Two-tailed)  Wilcoxon  Matched-pairs  Signed Rank  (Two-tailed)  Wilcoxon  Matched-pairs  Signed Rank  (Two-tailed)  Wilcoxon  Matched-pairs  Signed Rank  (Two-tailed) | 3 v 4  5 v 6  7 v 8 | <0.0001  <0.0001  0.0650 |
|  |  |  |  |  |  |  |

| Figure | Condition | # | Sample Size | Statistical  Test | Comparison | P-value |
| --- | --- | --- | --- | --- | --- | --- |
| 3f | Empty group –  Pre-stimulus | 1 | 26 | Two-way  ANOVA | 1 v 2 | 0.3336 |
|  | Empty group –  Stimulus  aIPg group –  Pre-stimulus  aIPg group –  Stimulus  LC10a group –  Pre-stimulus  LC10a group –  Stimulus  LC10bc group –  Pre-stimulus  LC10bc group –  Stimulus | 2  3  4  5  6  7  8 | 26  26  26  23  23  23  23 | (Stimulus x Genotype, P <0.0001; Stimulus, P <0.0001; Genotype, P <0.0001; Experiment, P <0.0001) | 3 v 4  5 v 6  7 v 8 | <0.0001  <0.0001  0.2478 |
|  |  |  |  |  |  |  |

**Supplementary Table 7: Genotypes for** **Figure 4c**

| Genotype | Group |
| --- | --- |
| *w^1118^; LexAop-TRPA1/ BPp65ADZp; 20XUAS-GtACR1-EYFP, R72C11-iLexA/ BPZpGALDBD* | aIPg > TrpA empty > GtACR (empty group) |
| *w^1118^; LexAop-TRPA1/ R59B10-p65ADZp; 20XUAS-GtACR1-EYFP, R72C11-iLexA/ R24A02-ZpGAL4DBD* | aIPg > TrpA IB112-SS1 > GtACR (IB112-SS1 group) |
| *w^1118^; LexAop-TRPA1/+; 20XUAS-GtACR1-EYFP, R72C11-iLexA/ R59B10-p65ADZp, R24A02-ZpGAL4DBD* | aIPg > TrpA IB112-SS2 > GtACR (IB112-SS2 group) |

**Supplementary Table 8: Sample sizes and Statistics for** **Figure 4c**

Analysis – Average percent of time spent performing aggressive behaviors. The pre-stimulus average was calculated with 15s before the first stimulus period and the full 30s pre-stimulus periods prior to each stimulus. The stimulus average was calculated from the three 30s continuous stimulus (9 mW/cm^2^) periods.

Each experiment is a biological replicate and data are combined from 5 batches from independent parental crosses. Negative controls were performed with each batch.

| Figure | Condition | # | Sample Size | Statistical  Test | Comparison | P-value |
| --- | --- | --- | --- | --- | --- | --- |
| 4c | Empty group –  Pre-stimulus | 1 | 22 | Wilcoxon  Matched-pairs | 1 v 2 | 0.3880 |
|  | Empty group –  Stimulus  IB112-SS1 group –  Pre-stimulus  IB112-SS1 group –  Stimulus  IB112-SS2 group –  Pre-stimulus  IB112-SS2 group –  Stimulus | 2  3  4  5  6 | 22  17  17  20  20 | Signed Rank  (Two-tailed)  Wilcoxon  Matched-pairs  Signed Rank  (Two-tailed)  Wilcoxon  Matched-pairs  Signed Rank  (Two-tailed) | 3 v 4  5 v 6 | 0.0005  <0.0001 |
|  |  |  |  |  |  |  |

| Figure | Condition | # | Sample Size | Statistical  Test | Comparison | P-value |
| --- | --- | --- | --- | --- | --- | --- |
| 4c | Empty group –  Pre-stimulus | 1 | 22 | Two-way  ANOVA | 1 v 2 | 0.2271 |
|  | Empty group –  Stimulus  IB112-SS1 group –  Pre-stimulus  IB112-SS1 group –  Stimulus  IB112-SS2 group –  Pre-stimulus  IB112-SS2 group –  Stimulus | 2  3  4  5  6 | 22  17  17  20  20 | (Stimulus x Genotype, P <0.0001; Stimulus, P <0.0001; Genotype, P <0.0001; Experiment, P <0.0001) | 3 v 4  5 v 6 | <0.0001  <0.0001 |
|  |  |  |  |  |  |  |

**Supplementary Table 9: Genotypes for Figure 5e**

| Genotype | Cells Activated/Recorded | Stable split name |
| --- | --- | --- |
| *w^1118^,13XLexAop2-CsChrimson- tdTomato / w^1118^; R72C11-iLexA/ VT061221-p65ADZp; pJFRC81-10XUAS-IVS-Syn21-GFP-p10 / R18C12-ZpGAL4DBD* | aIPg/TuTuA_1 | SS77402 |
| *w^1118^,13XLexAop2-CsChrimson- tdTomato / w; +/VT061221-p65ADZp; pJFRC81-10XUAS-IVS-Syn21-GFP-p10 / R18C12-ZpGAL4DBD* | None/TuTuA_1 | SS77402 |

**Supplementary Table 10: Genotypes for Figure 5f**

| Genotype | Cells Activated/Recorded | Stable split name |
| --- | --- | --- |
| *w^1118^,13XLexAop2-CsChrimson- tdTomato / w^1118^; R72C11-iLexA/R21H09-p65ADZp; pJFRC81-10XUAS-IVS-Syn21-GFP-p10 / R34C04-ZpGAL4DBD* | aIPg/TuTuA_2 | SS77462 |
| *w^1118^,13xLexAop2-CsChrimson- tdTomato / w^1118^; +/R21H09-p65ADZp; pJFRC81-10XUAS-IVS-Syn21-GFP-p10/ R34C04-ZpGAL4DBD* | None/TuTuA_2 | SS77462 |

**Supplementary Table 11: Genotypes for Figure 6a Left Panel**

| Genotype | Group |
| --- | --- |
| *w^1118^; LexAop-TRPA1/ BPp65ADZp; 20XUAS-GtACR1-EYFP, R72C11-iLexA/ BPZpGAL4DBD* | aIPg > TrpA empty > GtACR (empty group) |
| *w^1118^; LexAop-TRPA1/ VT061221-p65ADZp; 20XUAS-GtACR1-EYFP, R72C11-iLexA/ 18C12-ZpGAL4DBD* | aIPg > TrpA TuTuA_1 > GtACR (TuTuA_1 group) |

**Supplementary Table 12: Sample sizes and Statistics for Figure 6a Left Panel**

Left panel analysis – Average percent of time spent performing aggressive behaviors during 10s before each stimulus compared to the first 10s of three continuous stimulus (9 mW/cm^2^) periods.

Each experiment is a biological replicate and data are combined from 6 batches from independent parental crosses. Negative controls were performed with each batch.

| Figure | Condition | # | Sample Size | Statistical  Test | Comparison | P-value |
| --- | --- | --- | --- | --- | --- | --- |
| 6a  Left | Empty group –  Pre-stimulus | 1 | 23 | Wilcoxon  Matched-pairs | 1 v 2 | 0.4274 |
| Panel | Empty group –  Stimulus  TuTuA_1 group –  Pre-stimulus  TuTuA_1 group –  Stimulus | 2  3  4 | 23  24  24 | Signed Rank  (Two-tailed)  Wilcoxon  Matched-pairs  Signed Rank  (Two-tailed) | 3 v 4 | <0.0001 |
|  |  |  |  |  |  |  |

| Figure | Condition | # | Sample Size | Statistical  Test | Comparison | P-value |
| --- | --- | --- | --- | --- | --- | --- |
| 6a  Left | Empty group –  Pre-stimulus | 1 | 23 | Two-way  ANOVA | 1 v 2 | 0.4979 |
| Panel | Empty group –  Stimulus  TuTuA_1 group –  Pre-stimulus  TuTuA_1 group –  Stimulus | 2  3  4 | 23  24  24 | (Stimulus x Genotype, P <0.0001; Stimulus, P 0.0017; Genotype, P = 0.1258; Experiment, P <0.0001) | 3 v 4 | <0.0001 |
|  |  |  |  |  |  |  |

**Supplementary Table 13: Genotypes for Figure 6a Right Panel**

| Genotype | Group |
| --- | --- |
| *w^1118^,20XUAS-CsChrimson-mVenus/ w^1118^; LexAop-TRPA1/ BPp65ADZp; R72C11-iLexA/ BPZpGAL4DBD* | aIPg > TrpA empty > Chrimson (empty group) |
| *w^1118^,20XUAS-CsChrimson-mVenus/ w^1118^; LexAop-TRPA1/ R21H09-p65ADZp; R72C11-iLexA / R34C04-ZpGAL4DBD* | aIPg > TrpA TuTuA_2 > Chrimson (TuTuA_2 group) |

**Supplementary Table 14: Sample sizes and Statistics for Figure 6a Right Panel**

Right panel analysis – Average percent of time spent performing aggressive behaviors. The pre-stimulus average was calculated with 15s before the first stimulus period and the full 30s pre-stimulus periods prior to each stimulus. The stimulus average was calculated from the three 30s continuous stimulus (3 mW/cm^2^) periods.

Each experiment is a biological replicate and data are combined from 3 batches from independent parental crosses. Negative controls were performed with each batch.

| Figure | Condition | # | Sample Size | Statistical  Test | Comparison | P-value |
| --- | --- | --- | --- | --- | --- | --- |
| 6a  Right | Empty group –  Pre-stimulus | 1 | 19 | Wilcoxon  Matched-pairs | 1 v 2 | 0.8906 |
| Panel | Empty group –  Stimulus  TuTuA_2 group –  Pre-stimulus  TuTuA_2 group –  Stimulus | 2  3  4 | 19  20  20 | Signed Rank  (Two-tailed)  Wilcoxon  Matched-pairs  Signed Rank  (Two-tailed) | 3 v 4 | 0.0004 |
|  |  |  |  |  |  |  |

| Figure | Condition | # | Sample Size | Statistical  Test | Comparison | P-value |
| --- | --- | --- | --- | --- | --- | --- |
| 6a  Right | Empty group –  Pre-stimulus | 1 | 19 | Two-way  ANOVA | 1 v 2 | 0.9522 |
| Panel | Empty group –  Stimulus  TuTuA_2 group –  Pre-stimulus  TuTuA_2 group –  Stimulus | 2  3  4 | 19  20  20 | (Stimulus x Genotype, P = 0.0031; Stimulus, P = 0.0039; Genotype, P = 0.4278; Experiment, P <0.0001) | 3 v 4 | 0.0001 |
|  |  |  |  |  |  |  |

**Supplementary Table 15: Genotypes for Figure 6b**

| Genotype | Group |
| --- | --- |
| *w^1118^;+/ BPp65ADZp;20XUAS-GtACR1-EYFP/ BPZpGAL4DBD* | empty > GtACR (empty group) |
| *w^1118^;+/ R21H09-p65ADZp;20XUAS-GtACR1-EYFP/ R34C04-ZpGAL4DBD* | TuTuA_2 > GtACR (TuTuA_2 group) |

**Supplementary Table 16: Sample sizes and Statistics for Figure 6b**

Analysis – Average percent of time spent performing aggressive behaviors. The pre-stimulus average was calculated with 15s before the first stimulus period and the full 30s pre-stimulus periods prior to each stimulus. The stimulus average was calculated from the three 30s continuous stimulus (9 mW/cm^2^) periods.

Each experiment is a biological replicate and data are combined from 3 batches from independent parental crosses. Negative controls were performed with each batch.

| Figure | Condition | # | Sample Size | Statistical  Test | Comparison | P-value |
| --- | --- | --- | --- | --- | --- | --- |
| 6b | Empty group –  Pre-stimulus | 1 | 20 | Wilcoxon  Matched-pairs | 1 v 2 | 0.0215 |
|  | Empty group –  Stimulus  TuTuA_2 group –  Pre-stimulus  TuTuA_2 group –  Stimulus | 2  3  4 | 20  20  20 | Signed Rank  (Two-tailed)  Wilcoxon  Matched-pairs  Signed Rank  (Two-tailed) | 3 v 4 | <0.0001 |
|  |  |  |  |  |  |  |

| Figure | Condition | # | Sample Size | Statistical  Test | Comparison | P-value |
| --- | --- | --- | --- | --- | --- | --- |
| 6b | Empty group –  Pre-stimulus | 1 | 20 | Two-way  ANOVA | 1 v 2 | 0.4792 |
|  | Empty group –  Stimulus  TuTuA_2 group –  Pre-stimulus  TuTuA_2 group –  Stimulus | 2  3  4 | 20  20  20 | (Stimulus x Genotype, P <0.0001; Stimulus, P <0.0001; Genotype, P <0.0001; Experiment, P = 0.1810) | 3 v 4 | <0.0001 |
|  |  |  |  |  |  |  |

**Supplementary Table 17: Genotypes for Figure 6c**

| Genotype | Cells Imaged/Indicator | Stable split name |
| --- | --- | --- |
| *w^1118^;+/ VT061221-p65ADZp; 20xUAS-IVS-jGCaMP7f / R18C12-ZpGAL4DBD* | TuTuA_1/GCaMP7f | SS77402 |
| *w^1118^;+/ R21H09-p65ADZp ; 20XUAS-IVS-jGCaMP7f / R34C04-ZpAL4DBD* | TuTuA_2/GCaMP7f | SS77462 |

**Supplementary Table 18: Genotypes for Extended Data Figure 1b**

| Genotype | Group |
| --- | --- |
| *w^1118^; 20XUAS-CsChrimson-mVenus/ VT064565-p65ADZp; VT043699-ZpGAL4DBD* | aIPg > Chrimson (aIPg group) |
| *norpA^36^/ norpA^36^; 20XUAS-CsChrimson-mVenus/ BPp65ADZp; +/ BPZpGAL4DBD* | empty > Chrimson (empty group) |
| *norpA^36^/ norpA^36^; 20XUAS-CsChrimson-mVenus/ VT064565-p65ADZp; +/ VT043699-ZpGAL4DBD* | NorpA^-^ aIPg > Chrimson (NorpA aIPg group) |

**Supplementary Table 19: Sample sizes and Statistics for Extended Data Figure 1b**

Analysis – Average percent of time spent performing aggressive behaviors during the 30s before each stimulus prior compared to the 30s stimulus (3 mW/cm^2^) periods. Data from the low stimulus periods (1 mW/cm^2^) prior are not shown.

Each experiment is a biological replicate and data are representative of 2 independent batches with separate parental crosses. Negative controls were performed with each batch.

| Figure | Condition | # | Sample Size | Statistical  Test | Comparison | P-value |
| --- | --- | --- | --- | --- | --- | --- |
| S1b | aIPg group –  Baseline | 1 | 6 | Wilcoxon  Matched-pairs | 1 v 2 | 0.0312 |
|  | aIPg group –  Stimulus  NorpA Empty group –  Baseline  NorpA Empty group –  Stimulus  NorpA aIPg group –  Baseline  NorpA aIPg group –  Stimulus | 2  3  4  5  6 | 6  6  6  7  7 | Signed Rank  (Two-tailed)  Wilcoxon  Matched-pairs  Signed Rank  (Two-tailed)  Wilcoxon  Matched-pairs  Signed Rank  (Two-tailed) | 3 v 4  5 v 6 | 0.0625  0.3750 |
|  |  |  |  |  |  |  |

| Figure | Condition | # | Sample Size | Statistical  Test | Comparison | P-value |
| --- | --- | --- | --- | --- | --- | --- |
| S1b | aIPg group –  Pre-stimulus | 1 | 6 | Two-way  ANOVA | 1 v 2 | <0.0001 |
|  | aIPg group –  Stimulus  NorpA Empty group –  Pre-stimulus  NorpA Empty group –  Stimulus  NorpA aIPg group –  Pre-stimulus  NorpA aIPg group –  Stimulus | 2  3  4  5  6 | 6  6  6  7  7 | (Stimulus x Genotype, P <0.0001; Stimulus, P <0.0001; Genotype, P <0.0001; Experiment, P = 0.0058) | 3 v 4  5 v 6 | 0.7069  0.8883 |
|  |  |  |  |  |  |  |

**Supplementary Table 20: Genotypes for Extended Data Figure 2a – d**

| Genotype | Cells Imaged/Indicator | Stable split name |
| --- | --- | --- |
| *w^+^(DL)/ w^1118^; +/R28G03-p65ADZp;20XUAS-IVS-Syn21-GCaMP6f-p10/ VT027704-ZpGAL4DBD* | LC9/GCaMP6f | SS02662 |
| *w^+^(DL)/ w^1118^; +/ R35D04-p65ADZp; 20XUAS-IVS-Syn21-GCaMP6f-p10/ R22D06-ZpGAL4DBD* | LC10a/GCaMP6f | OL0019B |

**Supplementary Table 21: Genotypes for Extended Data Figure 2e – f**

| Genotype | Group |
| --- | --- |
| *Canton-S* | Courtship (Male-Female) |
| *w^1118^; LexAop-TRPA1/ BPp65ADZp; 20XUAS-GtACR1-EYFP, R72C11-iLexA/ BPZpGAL4DBD* (aIPg-LexA > TrpA empty-SS > GtACR) | Aggression (Female) |

**Supplementary Table 22: Genotypes for Extended Data Figure 3b – d**

| Genotype | Group |
| --- | --- |
| *w^1118^; LexAop-TRPA1/ BPp65ADZp; 20XUAS-GtACR1-EYFP, R72C11-iLexA/ BPZpGAL4DBD* | aIPg > TrpA empty > GtACR (empty group) |
| *w^1118^; LexAop-TRPA1/ VT064565-p65ADZp; 20XUAS-GtACR1-EYFP, R72C11-iLexA/ VT043699-ZpGAL4DBD* | aIPg > TrpA aIPg > GtACR (aIPg group) |
| *w^1118^; LexAop-TRPA1/ R35D04-p65ADZp; 20XUAS-GtACR1-EYFP, R72C11-iLexA/ R22D06-ZpGAL4DBD* | aIPg > TrpA LC10a > GtACR (LC10a group) |
| *w^1118^; LexAop-TRPA1/ R35D04-p65ADZp; 20XUAS-GtACR1-EYFP, R72C11-iLexA/ VT040747-ZpGAL4DBD* | aIPg > TrpA LC10bc > GtACR (LC10bc group) |

**Supplementary Table 23: Sample sizes and Statistics for Extended Data Figure 3b – d**

Analysis – Mean number of flies within 2 body lengths during aggressive behaviors performed during the three 30 s continuous stimulus (9 mW/cm^2^) periods.

Each experiment is a biological replicate and data are combined from 6 batches from independent parental crosses. Negative controls were performed with each batch.

| Figure | Condition | # | Sample Size | Statistical  Test | Comparison | P-value |
| --- | --- | --- | --- | --- | --- | --- |
| S3d | Empty group –  Stimulus | 1 | 26 | Kruskal-Wallis  (P <0.0001) | 1 v 2  1 v 3 | <0.0001  0.0048 |
|  | aIPg group –  Stimulus  LC10a group –  Stimulus  LC10bc group –  Stimulus | 2  3  4 | 26  23  23 | Dunn’s Multiple  Comparisons | 1 v 4  2 v 3  2 v 4  3 v 4 | 0.7467  0.7353  <0.0001  <0.0001 |
|  |  |  |  |  |  |  |

**Supplementary Table 24: Genotypes for Extended Data Figure 3e – g**

| Genotype | Group |
| --- | --- |
| *w^1118^; LexAop-TRPA1/ BPp65ADZp; 20XUAS-GtACR1-EYFP, R72C11-iLexA/ BPZpGLA4DBD* | aIPg > TrpA empty > GtACR (empty group) |
| *w^1118^; LexAop-TRPA1/ VT064565-p65ADZp; 20XUAS-GtACR1-EYFP, R72C11-iLexA/ VT043699-ZpGAL4DBD* | aIPg > TrpA aIPg > GtACR (aIPg group) |
| *w^1118^; LexAop-TRPA1/ R28G03-p65ADZp; 20XUAS-GtACR1-EYFP, R72C11-iLexA/ VT027704-ZpGAL4DBD* | aIPg > TrpA LC9 > GtACR (LC9 group) |
| *w^1118^ LexAop-TRPA1/R22H02-p65ADZp; 20XUAS-GtACR1-EYFP, R72C11-iLexA/R20G06-ZpGAL4DBD* | aIPg > TrpA LC11 > GtACR (LC11 group) |
| *w^1118^; LexAop-TRPA1/R26A03-p65ADZp; 20XUAS-GtACR1-EYFP, R72C11-iLexA/R24A02-ZpGAL4DBD* | aIPg > TrpA LC15 > GtACR (LC15 group) |

**Supplementary Table 25: Sample sizes and Statistics for Extended Data Figure 3e – g**

Analysis – Average percent of time spent performing aggressive behaviors. The pre-stimulus average was calculated with 15s before the first stimulus period and the full 30s pre-stimulus periods prior to each stimulus. The stimulus average was calculated from the three 30s continuous stimulus (9 mW/cm^2^) periods.

Each experiment is a biological replicate and data are combined from 8 batches from independent parental crosses. Negative controls were performed with each batch.

| Figure | Condition | # | Sample Size | Statistical  Test | Comparison | P-value |
| --- | --- | --- | --- | --- | --- | --- |
| S3g | Empty group –  Pre-stimulus | 1 | 28 | Wilcoxon  Matched-pairs | 1 v 2 | 0.9375 |
|  | Empty group –  Stimulus  LC9 group –  Pre-stimulus  LC9 group –  Stimulus  LC11 group –  Pre-stimulus  LC11 group –  Stimulus  LC15 group –  Pre-stimulus  LC15 group –  Stimulus | 2  3  4  5  6  7  8 | 28  15  15  23  23  18  18 | Signed Rank  (Two-tailed)  Wilcoxon  Matched-pairs  Signed Rank  (Two-tailed)  Wilcoxon  Matched-pairs  Signed Rank  (Two-tailed)  Wilcoxon  Matched-pairs  Signed Rank  (Two-tailed) | 3 v 4  5 v 6  7 v 8 | 0.0554  0.0024  0.4683 |
|  |  |  |  |  |  |  |

| Figure | Condition | # | Sample Size | Statistical  Test | Comparison | P-value |
| --- | --- | --- | --- | --- | --- | --- |
| S3g | Empty group –  Pre-stimulus | 1 | 28 | Two-way  ANOVA | 1 v 2 | 0.8744 |
|  | Empty group –  Stimulus  LC9 group –  Pre-stimulus  LC9 group –  Stimulus  LC11 group –  Pre-stimulus  LC11 group –  Stimulus  LC15 group –  Pre-stimulus  LC15 group –  Stimulus | 2  3  4  5  6  7  8 | 28  15  15  23  23  18  18 | (Stimulus x Genotype, P = 0.0148; Stimulus, P = 0.0168; Genotype, P = 0.0029; Experiment, P <0.0001) | 3 v 4  5 v 6  7 v 8 | 0.0246  0.0015  0.5298 |
|  |  |  |  |  |  |  |

**Supplementary Table 26: Genotypes for Extended Data Figure 5a**

| Genotype | Cells Activated/Recorded | Stable split name |
| --- | --- | --- |
| *w^1118^,13XLexAop2-CsChrimson- tdTomato / w^1118^; R72C11-iLexA/R59B10-p65ADZp; pJFRC81-10XUAS-IVS-Syn21-GFP-p10 /24A02-ZpGAL4DBD* | aIPg/IB112 | SS81529 |
| *w^1118^,13XLexAop2-CsChrimson- tdTomato / w^1118^; +/R59B10-p65ADZp; pJFRC81-10XUAS-IVS-Syn21-GFP-p10 /R24A02-ZpGAL4DBD* | None/IB112 | SS81529 |

**Supplementary Table 27: Genotypes for Extended Data Figure 5b**

| Genotype | Cells Activated/Imaged/Indicator | Stable split name |
| --- | --- | --- |
| *w^1118^,13XLexAop2-CsChrimson-tdTomato (attP18), 20XUAS-IVS-Syn21-opGCaMP6f-p10 (su(Hw)attP8); VT029314-LexA (JK22C)/ R59B10-p65ADZp; +/24A02-ZpGAL4DBD* | aIPg/IB112/GCaMP6f | SS81529 |
| *w^1118^,13xLexAop2-CsChrimson-tdTomato (attP18), 20xUAS-IVS-Syn21-opGCaMP6f-p10 (su(Hw)attP8); CyO/R59B10-p65ADZp; TM6B/R24A02-ZpGAL4DBD* | None/IB112/GCaMP6f | SS81529 |

**Supplementary Table 28: Genotypes for Extended Data Figure 5c – e**

| Genotype | Group |
| --- | --- |
| *w^1118^; LexAop-TRPA1/ BPp65ADZp; 20XUAS-GtACR1-EYFP, R72C11-iLexA/ BPZpGAL4DBD* | aIPg > TrpA empty > GtACR (empty group) |
| *w^1118^; LexAop-TRPA1/ VT064565-p65ADZp; 20XUAS-GtACR1-EYFP, R72C11-iLexA/ VT043699-ZpGAL4DBD* | aIPg > TrpA aIPg > GtACR (aIPg group) |
| *w^1118^; LexAop-TRPA1/ R59B10-p65ADZp; 20XUAS-GtACR1-EYFP, R72C11-iLexA/ 24A02-ZpGAL4DBD* | aIPg > TrpA IB112-SS1 > GtACR (IB112-SS1 group) |
| *w^1118^; LexAop-TRPA1/+; 20XUAS-GtACR1-EYFP, R72C11-iLexA/ R59B10-p65ADZp, R24A02-ZpGAL4DBD* | aIPg > TrpA IB112-SS2 > GtACR (IB112-SS2 group) |

**Supplementary Table 29: Genotypes for Extended Data Figure 7a – e**

| Genotype | Cells Activated/Imaged/Indicator | Stable split name |
| --- | --- | --- |
| *w^1118^,13XLexAop2-CsChrimson-tdTomato, 20XUAS-IVS-Syn21-opGCaMP6f- p10; +/ VT061221-p65ADZp;R 72C11-LexAi/R18C12-ZpGAL4DBD* | aIPg/TuTuA_1/GCaMP6f | SS77402 |
| *w^1118^,13XLexAop2-CsChrimson-tdTomato, 20XUAS-IVS-Syn21-opGCaMP6f-p10; +/R21H09-p65ADZp; R72C11-LexAi/R34C04-ZpGAL4DBD* | aIPg/TuTuA_2/GCaMP6f | SS77462 |
| *w^1118^,13XLexAop2-CsChrimson-tdTomato, 20XUAS-IVS-Syn21-opGCaMP6f-p10; VT029314-LexA/ VT061221-p65ADZp; +/R18C12-ZpGAL4DBD* | LC10a/TuTuA_1/GCaMP6f | SS77402 |
| *w^1118^,13XLexAop2-CsChrimson-tdTomato, 20XUAS-IVS-Syn21-opGCaMP6f-p10; VT029314-LexA/R21H09-p65ADZp; +/R34C04-ZpGAL4DBD* | LC10a/TuTuA_2/GCaMP6f | SS77462 |
| *w^1118^,13XLexAop2-CsChrimson-tdTomato, 20XUAS-IVS-Syn21-opGCaMP6f-p10; CyO/ VT061221-p65ADZp; TM6B/R18C12-ZpGAL4DBD* | None/TuTuA_1/GCaMP6f | SS77402 |
| *w^1118^,13XLexAop2-CsChrimson-tdTomato, 20XUAS-IVS-Syn21-opGCaMP6f-p10; CyO/R21H09-p65ADZp; TM6B/R34C04-ZpGAL4DBD* | None/TuTuA_2/GCaMP6f | SS77462 |

**Supplementary Table 30: Genotypes for Extended Data Figure 7g – k**

| Genotype | Cells Activated/Recorded | Stable split name |
| --- | --- | --- |
| *w^1118^,20XUAS-CSChrimson-tdTomato/ w^1118^; pJFRC57-13XLexAop2-IVS-GFP-p10/+; SL96683/R35C10-p65ADZp, R71A09-ZpGAL4DBD* | pC1d/TuTuA_2 | SS56987 |
| *w^1118^,20XUAS-CSChrimson-tdTomato/ w^1118^; pJFRC57-13XLexAop2-IVS-GFP-p10/+; SL96683/R35C10-p65ADZp, R60G08-ZpGAL4DBD* | pC1e/TuTuA_2 | SS59336 |
| *w^1118^,20XUAS-CSChrimson-tdTomato/ w^1118^; pJFRC57-13XLexAop2-IVS-GFP-p10/ VT025602-p65ADZp; SL96683/ VT002064-ZpGdbd* | pC1de/TuTuA_2 | SS43274 |
| *w^1118^,20XUAS-CSChrimson-tdTomato/ w^1118^; pJFRC57-13XLexAop2-IVS-GFP-p10/ BPp65ADZp; SL96683/BPZpGAL4DBD* | empty/TuTuA_2 | Empty-SS |

**Supplementary Table 31: Sample sizes and Statistics for Extended Data Figure 7g – k**

Analysis – Latency after stimulus onset (ms).

| Figure | Condition | # | Sample Size | Statistical  Test | Comparison | P-value |
| --- | --- | --- | --- | --- | --- | --- |
| S7k | Activated aIPg  Record TuTuA_1 | 1 | 16 | Kruskal-  Wallis  (P < 0.0001) | 1 v 2  1 v 3  1 v 4 | 0.0005  0.0570  0.0009 |
|  | Activated aIPg  Record TuTuA_2  Activated pC1e  Record TuTuA_2  Activated pC1d/e  Record TuTuA_2 | 2  3  4 | 16  6  7 | Dunn’s Multiple  Comparisons | 2 v 3  2 v 4  3 v 4 | >0.9999  >0.9999  >0.9999 |
|  |  |  |  |  |  |  |

**Supplementary Table 32: Genotypes for Extended Data Figure 8a, e Top Panel**

| Genotype | Group |
| --- | --- |
| *w^1118^; LexAop-TRPA1/ BPp65ADZp; 20XUAS-GtACR1-EYFP, R72C11-iLexA/ BPZpGAL4DBD* | aIPg > TrpA empty > GtACR (empty group) |
| *w^1118^; LexAop-TRPA1/ VT061221-p65ADZp; 20XUAS-GtACR1-EYFP, R72C11-iLexA/ 18C12-ZpGAL4DBD* | aIPg > TrpA TuTuA_1 > GtACR (TuTuA_1 group) |

**Supplementary Table 33: Genotypes for Extended Data Figure 8b, e Bottom Panel**

| Genotype | Group |
| --- | --- |
| *w^1118^,20XUAS-CsChrimson-mVenus/ w^1118^; LexAop-TRPA1/ BPp65ADZp; R72C11-iLexA/ BPZpGAL4DBD* | aIPg > TrpA empty > Chrimson (empty group) |
| *w^1118^,20XUAS-CsChrimson-mVenus/ w^1118^; LexAop-TRPA1/ R21H09-p65ADZp; R72C11-iLexA / R34C04-ZpGAL4DBD* | aIPg > TrpA TuTuA_2 > Chrimson (TuTuA_2 group) |

**Supplementary Table 34: Genotypes for Extended Data Figure 8c – d**

| Genotype | Group |
| --- | --- |
| *w^1118^,20XUAS-CsChrimson-mVenus/ w^1118^; LexAop-TRPA1/ BPp65ADZp; R72C11-iLexA/ BPZpGAL4DBD* | aIPg > TrpA empty > Chrimson (empty group) |
| *w^1118^,20XUAS-CsChrimson-mVenus/ w^1118^; LexAop-TRPA1/R21H09-p65ADZp; R72C11-iLexA / VT016645-ZpGAL4DBD* | aIPg > TrpA TuTuA_2-SS2 > Chrimson (TuTuA_2-SS2 group) |

**Supplementary Table 35: Sample sizes and Statistics for Extended Data Figure 8c – d**

Analysis – Average percent of time spent performing aggressive behaviors. The pre-stimulus average was calculated with 15s before the first stimulus period and the full 30s pre-stimulus periods prior to each stimulus. The stimulus average was calculated from the three 30s continuous stimulus (3 mW/cm^2^) periods.

Each experiment is a biological replicate and data are combined from 3 batches from independent parental crosses. Negative controls were performed with each batch.

| Figure | Condition | # | Sample Size | Statistical  Test | Comparison | P-value |
| --- | --- | --- | --- | --- | --- | --- |
| S8d | Empty group –  Pre-stimulus | 1 | 12 | Wilcoxon  Matched-pairs | 1 v 2 | 0.3804 |
|  | Empty group –  Stimulus  TuTuA_2-SS2 group –  Pre-stimulus  TuTuA_2-SS2 group –  Stimulus | 2  3  4 | 12  13  13 | Signed Rank  (Two-tailed)  Wilcoxon  Matched-pairs  Signed Rank  (Two-tailed) | 3 v 4 | 0.0012 |
|  |  |  |  |  |  |  |

| Figure | Condition | # | Sample Size | Statistical  Test | Comparison | P-value |
| --- | --- | --- | --- | --- | --- | --- |
| S8d | Empty group –  Pre-stimulus | 1 | 12 | Two-way  ANOVA | 1 v 2 | 0.4152 |
|  | Empty group –  Stimulus  TuTuA_2-SS2 group –  Pre-stimulus  TuTuA_2-SS2 group –  Stimulus | 2  3  4 | 12  13  13 | (Stimulus x Genotype, P = 0.0080; Stimulus, P = 0.0004; Genotype, P = 0.0181; Experiment, P <0.0001) | 3 v 4 | <0.0001 |
|  |  |  |  |  |  |  |

**Supplementary Table 36: Genotypes for Extended Data Figure 8f**

| Genotype | Group |
| --- | --- |
| *w^1118^;+/ BPp65ADZp;20XUAS-GtACR1-EYFP/ BPZpGAL4DBD* | empty > GtACR (empty group) |
| *w^1118^;+/ R21H09-p65ADZp;20XUAS-GtACR1-EYFP/ R34C04-ZpGAL4DBD* | TuTuA_2 > GtACR (TuTuA_2 group) |

**Supplementary Table 37: Genotypes for Extended Data Figure 9**

| Genotype | Cells Recorded | Stable split name |
| --- | --- | --- |
| *w^1118^;+/VT061221-p65ADZp; pJFRC81-10XUAS-IVS-Syn21-GFP-p10 / R18C12-ZpGAL4DBD* | TuTuA_1 | SS77402 |
| *w^1118^;+/R21H09-p65ADZp; pJFRC81-10XUAS-IVS-Syn21-GFP-p10 / R34C04-ZpGAL4DBD* | TuTuA_2 | SS77462 |
