# Supplementary material for: Social state alters vision using three circuit mechanisms in Drosophila
Source: Nature. 2024 Nov 20;637(8046):646–53. doi: 10.1038/s41586-024-08255-6 (PMC11735400; doi:10.1038/s41586-024-08255-6)
Supplement: Supplementary file 2 — Reporting Summary [file 41586_2024_8255_MOESM2_ESM.pdf]

nature portfolio

Corresponding author(s): Gerald M. Rubin

Last updated by author(s): Aug 27, 2024

Reporting Summary

Nature Portfolio wishes to improve the reproducibility of the work that we publish. This form provides structure for consistency and transparency in reporting. For further information on Nature Portfolio policies, see our Editorial Policies and the Editorial Policy Checklist.

Statistics

For all statistical analyses, confirm that the following items are present in the figure legend, table legend, main text, or Methods section.

n/a	Confirmed	
☐	☒	The exact sample size (*n*) for each experimental group/condition, given as a discrete number and unit of measurement
☐	☒	A statement on whether measurements were taken from distinct samples or whether the same sample was measured repeatedly
☐	☒	The statistical test(s) used AND whether they are one- or two-sided<br>*Only common tests should be described solely by name; describe more complex techniques in the Methods section.*
☐	☒	A description of all covariates tested
☐	☒	A description of any assumptions or corrections, such as tests of normality and adjustment for multiple comparisons
☐	☒	A full description of the statistical parameters including central tendency (e.g. means) or other basic estimates (e.g. regression coefficient) AND variation (e.g. standard deviation) or associated estimates of uncertainty (e.g. confidence intervals)
☐	☒	For null hypothesis testing, the test statistic (e.g. *F*, *t*, *r*) with confidence intervals, effect sizes, degrees of freedom and *P* value noted<br>*Give P values as exact values whenever suitable.*
☒	☐	For Bayesian analysis, information on the choice of priors and Markov chain Monte Carlo settings
☒	☐	For hierarchical and complex designs, identification of the appropriate level for tests and full reporting of outcomes
☒	☐	Estimates of effect sizes (e.g. Cohen's *d*, Pearson's *r*), indicating how they were calculated

Our web collection on statistics for biologists contains articles on many of the points above.

Software and code

Policy information about availability of computer code

Data collection	For behavioral experiments, female aggression experiments were recorded from above using a camera (USB 3.1 Blackfly S, Monochrome Camera; Point Gray, Richmond, Canada) with an 800 nm long pass filter (B and W filter; Schneider Optics, Hauppauge, NY) at 170 frames per second and 1024 × 1024 pixel resolution. Animals were monitored during behavioral analysis using previously described and published BIAS software and MATLAB R2019a scripts (MathWorks). Male courtship experiments and animal trajectories collected during imaging were performed as detailed in Hindmarsh Sten et al. (2021).
Data analysis	Behavioral and imaging experiments were analyzed using MATLAB R2019a (MathWorks) and electrophysiological recordings were analyzed using pClamp (Clampfit 11.3). MATLAB (MathWorks) and GrapPad Prism 10 were used for statistics and data visualization.

For manuscripts utilizing custom algorithms or software that are central to the research but not yet described in published literature, software must be made available to editors and reviewers. We strongly encourage code deposition in a community repository (e.g. GitHub). See the Nature Portfolio guidelines for submitting code & software for further information.

Data

Policy information about availability of data

All manuscripts must include a data availability statement. This statement should provide the following information, where applicable:

- Accession codes, unique identifiers, or web links for publicly available datasets
- A description of any restrictions on data availability
- For clinical datasets or third party data, please ensure that the statement adheres to our policy

All data underlying figures is available as supplementary materials (see Source Data) and code is provided in figshare (DOI: 10.25378/janelia.26849083) and github (https://github.com/ceschretter/SocialState2024_Code). Raw data is available upon request from the corresponding author.

Research involving human participants, their data, or biological material

Policy information about studies with human participants or human data. See also policy information about sex, gender (identity/presentation), and sexual orientation and race, ethnicity and racism.

Reporting on sex and gender	N/A
Reporting on race, ethnicity, or other socially relevant groupings	N/A
Population characteristics	N/A
Recruitment	N/A
Ethics oversight	N/A

Note that full information on the approval of the study protocol must also be provided in the manuscript.

Field-specific reporting

Please select the one below that is the best fit for your research. If you are not sure, read the appropriate sections before making your selection.

☒ Life sciences ☐ Behavioural & social sciences ☐ Ecological, evolutionary & environmental sciences

For a reference copy of the document with all sections, see nature.com/documents/nr-reporting-summary-flat.pdf

Life sciences study design

All studies must disclose on these points even when the disclosure is negative.

Sample size	No statistical methods were used to pre-determine sample size. Sample size was based on previous literature in the field.
Data exclusions	For tethered courtship experiments, only experiments during which animals exhibited courtship towards the visual targets were included for analysis. For all other behavioral and imaging experiments, data was only excluded in the event of acquisition error or data corruption. In Extended Data Figure 5b and 7a – e, outlier samples with very low intensities or those whose intensity randomly fluctuated were excluded from the analysis.
Replication	All attempts at replication were successful. Biological replicates completed at separate times using different parental crosses were performed for each of the behavioral experiments. Behavioral data are representative of at least two independent biological repeats. All functional imaging and electrophysiological recordings were replicated across at least three separate animals.
Randomization	As controls were performed within animals, no randomization was performed. This was not relevant for this study as all measurements of behavior and cell activity were quantitative assessments with no treatment groups.
Blinding	Experimenters were not blinded as all data acquisition and analysis was automated.

Reporting for specific materials, systems and methods

We require information from authors about some types of materials, experimental systems and methods used in many studies. Here, indicate whether each material, system or method listed is relevant to your study. If you are not sure if a list item applies to your research, read the appropriate section before selecting a response.

Materials & experimental systems

n/a	Involved in the study	
☐	☒	Antibodies
☒	☐	Eukaryotic cell lines
☒	☐	Palaeontology and archaeology
☐	☒	Animals and other organisms
☒	☐	Clinical data
☒	☐	Dual use research of concern
☒	☐	Plants

Methods

n/a	Involved in the study	
☒	☐	ChIP-seq
☒	☐	Flow cytometry
☒	☐	MRI-based neuroimaging

Antibodies

Antibodies used	Primary antibodies used were mouse nc82 (Developmental Studies Hybridoma Bank, nc82-s) and rabbit polyclonal α-GFP (Life Technologies, A11122). Secondary antibodies used were AF568 Goat α-Mouse (Life Technologies, A11031) and AF488 Goat α-Rabbit (Life Technologies, A11034).
Validation	All antibodies for this study have been used and validated previously (https://www.janelia.org/project-team/flylight/protocols).

Animals and other research organisms

Policy information about studies involving animals; ARRIVE guidelines recommended for reporting animal research, and Sex and Gender in Research

Laboratory animals	All flies used in behavioral and functional analysis were between 3 – 10 days post eclosion. Images of brains are males and females and are indicated on the figure and figure legend. Please refer to the methods and resources table for additional description of research animals.
Wild animals	No wild animals were used in this study.
Reporting on sex	Males and females were used throughout the study and carefully considered during the study design. For experiments examining female aggression, only females were used as the cell types being studied differ between males and females as does the line expression. For experiments examining male courtship behavior, males and females were used. Behavioral analysis of the male was performed to compare across sexes. Sex in this study is reported in each figure legend as well as throughout the text.
Field-collected samples	No field-collected animals were used in this study.
Ethics oversight	No ethical approval was required as all experiments were performed in Drosophila melanogaster.

Note that full information on the approval of the study protocol must also be provided in the manuscript.

Plants

Seed stocks	*Report on the source of all seed stocks or other plant material used. If applicable, state the seed stock centre and catalogue number. If plant specimens were collected from the field, describe the collection location, date and sampling procedures.*
Novel plant genotypes	*Describe the methods by which all novel plant genotypes were produced. This includes those generated by transgenic approaches, gene editing, chemical/radiation-based mutagenesis and hybridization. For transgenic lines, describe the transformation method, the number of independent lines analyzed and the generation upon which experiments were performed. For gene-edited lines, describe the editor used, the endogenous sequence targeted for editing, the targeting guide RNA sequence (if applicable) and how the editor was applied.*
Authentication	*Describe any authentication procedures for each seed stock used or novel genotype generated. Describe any experiments used to assess the effect of a mutation and, where applicable, how potential secondary effects (e.g. second site T-DNA insertions, mosiacism, off-target gene editing) were examined.*
